# The Trehalose Phosphotransferase System (PTS) in *E. coli* W Can Transport Low Levels of Sucrose that Are Sufficient to Facilitate Induction of the *csc* Sucrose Catabolism Operon

**DOI:** 10.1371/journal.pone.0088688

**Published:** 2014-02-28

**Authors:** Jennifer A. Steen, Nina Bohlke, Claudia E. Vickers, Lars K. Nielsen

**Affiliations:** Australian Institute for Bioengineering and Nanotechnology (AIBN), The University of Queensland, Brisbane, Queensland, Australia; Louisiana State University and A & M College, United States of America

## Abstract

Plasticity in substrate acceptance is a well-characterised phenomenon for disaccharide transporters. Sucrose, a non-reducing disaccharide, is usually metabolised *via* either the permease-mediated chromosomally-encoded sucrose catabolism (*csc*) regulon or the sucrose phosphotransferase system (PTS). *E. coli* W is a fast-growing strain which efficiently utilises sucrose at concentrations above 1% *via* the *csc* regulon. To examine if sucrose could be metabolised *via* other routes, a library of transposon mutants was generated and screened on 0.2% sucrose. One mutant identified from this library had an insertion in the repressor for the regulon controlling catabolism of the disaccharide trehalose (*treR*). A series of mutants was constructed to elucidate the mechanism of sucrose utilization in the *treR* insertion strain. Analysis of these mutants provided evidence that deletion of TreR enables uptake of sucrose *via* TreB, an enzyme II protein required for PTS-mediated uptake of trehalose. Once inside the cell, this sucrose is not processed by the TreC hydrolase, nor is it sufficient for growth of the strain. QRT-PCR analysis showed that levels of *cscA* (invertase) transcript increased in the WΔ*treR* mutant relative to the wild-type strain when grown under low sucrose conditions. This result suggests that the intracellular sucrose provided by TreB can facilitate de-repression of the *csc* regulon, leading to increased gene expression, sucrose uptake and sucrose utilization in the *treR* mutant.

## Introduction

Sucrose shows strong potential as an industrial feedstock for *E. coli*-based bioprocesses [1]–[3]; in addition, sucrose utilisation is prevalent in pathogenic *E. coli* strains [4], [5]. Approximately 50% of wild-type *Escherichia coli* strains can metabolize sucrose [6] and in clinical isolates of enteropathogenic *E. coli* (EPEC) this can rise to 90% [4], [5]. Two mechanisms for sucrose metabolism have been described in strains of *E. coli*: the sucrose phosphotransferase system (PTS; [7]–[9] and the chromosomally-located sucrose catabolose (*csc*) regulon. The *csc* regulon was originally identified in *E. coli* EC3132 [1], [10], [11] but it has also been identified in the genomes of enterohaemorrhagic *E. coli* (EHEC), enterotoxigenic *E. coli* (ETEC) [12]–[15] and the non-pathogenic strain W [1], [16].

In the *csc* system, sucrose is imported into the cell by the permease, CscB [17]. This gene is essential for sucrose utilisation, and no other gene can provide sufficient levels of sucrose transport for growth on sucrose in a Δ*cscB* knockout [18]. Once in the cell, the sucrose is hydrolysed into fructose and glucose by the essential invertase, CscA [19]. The *cscA* gene is also essential for sucrose utilisation [18]. A fructokinase (CscK) phosphorylates the fructose moeity [11]. Sucrose utilization is tightly regulated, requiring both the absence of glucose and the presence of sucrose to allow for full induction of the regulon. In the presence of glucose, the regulon is under catabolite repression [20]–[22]. Operons under catabolite repression are activated when a complex of cyclic AMP (cAMP) and cAMP receptor protein A (CrpA) bind to the promoter [10]. The formation of this complex only occurs in the absence of glucose. Even in the absence of glucose, often these operons remain poorly expressed until such time as the cognate repressor is de-repressed *via* the action of an inducer. Repression of the divergently expressed *cscA* and *cscKB* operons by the regulator protein CscR is thought to occur *via* the *cscA* and *cscKB* operator binding sites located within the intergenic region between the two operons [10]. Under typical conditions, induction of the regulon occurs when the repressor binds to an inducer, blocking access to the operator binding site. The inducer of the *csc* regulon has not yet been experimentally determined; however, based on similar systems [23]–[25] it is likely to be either the sucrose or a product of the metabolism of sucrose (glucose/fructose/other). Unusually, the *csc* regulon requires relatively high (>1%) sucrose concentrations for de-repression [2], [11], [16]. Deletion/truncation of the *cscR* gene results in de-repression of the regulon, allowing sucrose utilisation at low concentrations [2], [11], . Mutations in *cscB* also confer growth on low sucrose concentrations [26], presumably *via* increased transport rates and subsequent increased in intracellular sucrose resulting in induction of the operon.

Although the *csc* regulon is highly conserved, notable variation in the sucrose utilisation phenotype has been observed, especially among EPEC strains [5]. For example, strains from serotypes O157:H7 and O55:H7 have been described as “slow” sucrose fermenters as they produce only light pink colonies following growth on MacConkey agar supplemented with 1% sucrose [5]. Furthermore, when laboratory strains are engineered to use sucrose by over-expressing *csc* genes, widely varying growth rates (from doubling times of many hours to growth rates similar to those observed on glucose) are observed [10], [11], [27]–[30]. Large variations in growth rate upon introduction of the same genes imply a strain-specific limitation in either sucrose utilisation or induction, and suggest that genes outside of the *csc* regulon contribute to the efficiency of sucrose utilisation/induction. To examine this possibility, we used *E. coli* W, a non-pathogenic strain that utilises sucrose *via* the *csc* regulon and does not have a sucrose PTS [1], as an experimental system. Although *E. coli* W is capable of growing very quickly on sucrose [31], sucrose metabolism is repressed at concentrations below ∼1% [2], [16]. A transposon mutagenesis library was developed in this strain and screened on low (0.2%) sucrose.

Several strains with altered sucrose utilisation phenotypes were identified; one of these had an insertion in the trehalose repressor gene, *TreR*. Using targeted mutagenesis and qRT-PCR, we examined the mechanism of sucrose utilization in the WΔ*treR* mutant. Our data suggest that sucrose can be transported by the trehalose PTS transporter, TreB. The transported sucrose is insufficient for cell growth, but can facilitate induction of the *csc* operon and subsequent growth *via* cscB-mediated sucrose uptake. To our knowledge, this is the first time that TreB has been shown to capable of sucrose transport.

## Materials and Methods

### Bacterial strains, media and growth conditions

Bacterial strains and plasmids are shown in Table 1. *E. coli* W (NCIMB 8666) was purchased from the National Collection of Industrial, Food and Marine Bacteria (NCIMB) (Aberdeen, UK). For general cloning and maintenance, *E. coli* strains were grown in LB medium [32]. For sugar utilization experiments, M9 minimal medium [32] supplemented with thiamine (1 mg/L) was used and supplemented with either 2 g/l sucrose (M9S2), 20 g/l sucrose (M9S20), or 1% w/v glycerol (M9Gly). Ampicillin (100 µg/mL), kanamycin (50 µg/mL), and/or chloramphenicol (25 µg/mL) were included in media where appropriate.

**Table 1:** *E. coli* strains and plasmids used in this study.

Strain	Relevant genotype or phenotype characteristics	Source
W	Wild-type *csc*+	NCIMB
WΔcscR	WΔ*cscR*::FRT	[2]
Tn2	W *appC::*Tn*5* (Kan^R^)	This study
Tn9	W *cyaA::*Tn*5* (Kan^R^)	This study
Tn32	W *mhpR::*Tn*5* and *cscB* L363F mutation (Kan^R^)	This study
Tn33	W *mutS::*Tn*5* (Kan^R^)	This study
Tn34	W with unknown Tn*5* insertion site and *cscB* L51F mutation (Kan^R^)	This study
Tn51	W with unknown Tn*5* insertion site and *cscB* L363F mutation (Kan^R^)	This study
Tn52	W with unknown Tn*5* insertion site and *cscB* F371V mutation Kan^R^	This study
Tn53	W with unknown Tn*5* insertion site and *cscR* Q57* mutation (Kan^R^)	This study
Tn54	W *pi349::*Tn*5* (Kan^R^)	This study
Tn56	W with unknown Tn*5* insertion site and *cscB* L51F mutation (Kan^R^)	This study
Tn57	W with unknown Tn*5* insertion site and *cscB* C327G mutation (Kan^R^)	This study
Tn58	W with unknown Tn*5* insertion site and *cscB* L51F mutation (Kan^R^)	This study
Tn61	W *treR::*Tn*5* (Kan^R^)	This study
Tn63	W *gspK::*Tn*5* (Kan^R^)	This study
Tn65	W *gltS::*Tn*5* (Kan^R^)	This study
Tn68	W *hyp::*Tn*5* (Kan^R^)	This study
Tn69	W with Tn5 insertion mutations and *cscKB* operator binding site mutation (Kan^R^)	This study
Tn84	W *truA::*Tn*5* (Kan^R^)	This study
Tn86	W with Tn5 insertion mutations and *cscB* L363F mutation (Kan^R^)	This study
Tn87	W *treR::*Tn*5* (Kan^R^)	This study
WΔgltS 1	WΔ*gltS*::FRT	This study
WΔgltS 2	WΔ*gltS*::FRT	This study
WΔgltS 3	WΔ*gltS*::FRT	This study
WΔ*gspK* 1	WΔ*gspK*::FRT	This study
WΔ*gspK* 2	WΔ*gspK*::FRT	This study
WΔ*gspK* 3	WΔ*gspK*::FRT	This study
WΔ*mphR* 1	WΔ*mphR*::FRT	This study
WΔ*mphR* 2	WΔ*mphR*::FRT	This study
WΔ*mphR* 3	WΔ*mphR*::FRT	This study
WΔ*treR* 1	WΔ*treR*::FRT	This study
WΔ*treR* 2	WΔ*treR*::FRT	This study
WΔ*treR* 3	WΔ*treR*::FRT	This study
WΔtreRΔtreB 1	WΔ*treR*::FRT Δ*treB*::FRT-*cat*-FRT. Parent: WΔ*treR* 1	This study
WΔtreRΔtreB 2	WΔ*treR*::FRT Δ*treB*::FRT-*cat*-FRT. Parent: WΔ*treR* 2	This study
WΔtreRΔtreB 3	WΔ*treR*::FRT Δ*treB*::FRT-*cat*-FRT. Parent: WΔ*treR* 3	This study
WΔtreRΔtreC 1	WΔ*treR*::FRT Δ*treC*::FRT-*cat*-FRT. Parent: WΔ*treR* 1	This study
WΔtreRΔtreC 3	WΔ*treR*::FRT Δ*treC*::FRT-*cat*-FRT. Parent: WΔ*treR* 3	This study
WΔtreRΔcscA 1	WΔ*treR*::FRT Δ *cscA*::FRT-*cat*-FRT. Parent: WΔ*treR* 1	This study
WΔtreRΔcscA 2	WΔ*treR*::FRT Δ*cscA*::FRT-*cat*-FRT. Parent: WΔ*treR* 2	This study
WΔtreRΔcscA 3	WΔ*treR*::FRT Δ *cscA*::FRT-*cat*-FRT. Parent: WΔ*treR* 3	This study
WΔtreRΔcscB 1	WΔ*treR*::FRT Δ*cscB*::FRT-*cat*-FRT. Parent: WΔ*treR* 1	This study
WΔtreRΔcscB 2	WΔ*treR*::FRT Δ*cscB*::FRT-*cat*-FRT. Parent: WΔ*treR* 2	This study
WΔtreRΔcscB 3	WΔ*treR*::FRT Δ*cscB*::FRT-*cat*-FRT. Parent: WΔ*treR* 3	This study

*csc* ^LS+^: positive growth on low sucrose (2 g/l).

Amp^R^, ampillicin resistance (100 µg/mL).

Kan^R^, kanamycin resistance (50 µg/mL).

Chl^R^, chloramphenicol resistance (40 µg/mL).

### General molecular biology

General molecular biology techniques were performed according to standard protocols [32]. Genomic DNA extraction was performed using either the Ultraclean® Microbial DNA Isolation kit (MO BIO Laboratories, Inc, CA) or the RBC Genomic Extraction kit (RBC bioscience, Taiwan) as per the manufacturers' instructions. Oligonucleotide primers used in this study are shown in Table 2.

**Table 2:** Primers used in this study.

Name	Sequence (5′ – 3′)	Application
JSP07	ACCTACAACAAAGCTCTCATCAACCGTGGC	PCR/Sequencing *
cscA_F2	ATGTCAGCCAAAGTATGGGTTTTAGG	PCR/Sequencing
cscA_R2	TTAACCCAGTAGCCAGAGTGC	PCR/Sequencing
cscA_R3	CAGATCAGGCCGTTTGGATC	PCR/Sequencing
cscB_F2	ATGGCACTGAATATTCCATTCAGA	PCR/Sequencing
cscB_R2	CTATATTGCTGAAGGTACAGGCGT	PCR/Sequencing
cscK_R3	GACTCCCTCAGTTAGCAGCG	PCR/Sequencing
cscK_F1	GCCGGGTTACTCACAGGTCTG	PCR/Sequencing
cscK_R1	TTCGCCGTTACTGCAAGCGCT	PCR/Sequencing
cscR_F3	CAGCTTCCACATGACATTATCG	PCR/Sequencing
cscOP_F1c	GCTCTAGAGGATACTGGGCGATGAGCGAG	PCR/Sequencing
cscOP_Rc	GCTCTAGACGACAATGTCCTGGAAATCAGC	PCR/Sequencing
cscR_R2	TCAGGTGGAACAACGGATC	PCR/Sequencing
cscR_F1	GTAACGATCGCGCAGCCTTTGTGG	PCR/Sequencing
glts_F	CTGCGCGATGAAGTATGACG	PCR/Sequencing
glts_R	GTTTCCCATACACAGAGCAC	PCR/Sequencing
gspK_F	CTCATGCAGCAAACGATGAG	PCR/Sequencing
gspK_R	AGCAACGTCAGACCAGATAC	PCR/Sequencing
mphR_F	CGTTTTCAGGTGCAAGGTCA	PCR/Sequencing
mphR_R	GCGACATCGTATAGCGTTAC	PCR/Sequencing
treR_F	GGCAATGCGCACTTAAGGAC	PCR/Sequencing
treR_R	CTGCAGAATATATTGCGCGG	PCR/Sequencing
treB_F	CCTGATCGTTTCCTGAACGA	PCR/Sequencing
treB_R	GTCATTACGTTATTCCTGCAAA	PCR/Sequencing
treC_F	CCTCGTTTATCTATCAGCGG	PCR/Sequencing
treC_R	CAACATGCTGACAGACAAAAC	PCR/Sequencing
gltS KO_F	ATGTTTCATCTCGATACTTTAGCAACGCTTGTTGCCGCAACGCTGACGTTGTGTAGGCTGGAGCTGCTTC	Mutagenesis
glts KO_R	TTAACCGGCAAAAATCGGCAACATTAAATACAGCTTAATCACCAGCGCATCATATGAATATCCTCCTTAG	Mutagenesis
gspK KO_F	ATGATCACCTCACCACCAAAACGCGGAATGGCACTGGTCGTGGTGCTGGTGTGTAGGCTGGAGCTGCTTC	Mutagenesis
gspK KO_R	TCACTCACTTTCTCCTGTCTGATGCCAGAGAACCGAAAAGTGTTGTGGGCCATATGAATATCCTCCTTAG	Mutagenesis
mphR KO_F	ATGATTTTTTATTGTGCGCTCAGTATAGGAAGGGTGTTTTCGGCTACAATGTGTAGGCTGGAGCTGCTTC	Mutagenesis
mphR KO_R	TTAACGCAAATGCACGCCGCTTCGCCGTCCGGCCACCAGAATAGCCTGCGCATATGAATATCCTCCTTAG	Mutagenesis
treR KO_F	AGGATGCAAAATCGGCTGACCATCAAAGACATCGCACGCTTAAGCGGCGTGTGTAGGCTGGAGCTGCTTC	Mutagenesis
treR KO_R	GATGATGATTTGTTGCGGTTCGCTGCGCCCGGTTACCTGTGCGATCAATTCATATGAATATCCTCCTTAG	Mutagenesis
treB KO_F	TGATGAGCAAAATAAACCAAACGGATATCGATCGGTTGATTGAACTGGTCACATATGAATATCCTCCTTAG	Mutagenesis
treB KO_R	AACAATATCCAGCGTGCCCAGGCGGTATTTCCGCTGATAGATAAACGAGGAGTGTAGGCTGGAGCTGCTTC	Mutagenesis
treC KO_F	ACTAATCTTCCCCACTGGTGGCAAAACGGCGTTATCTACCAGATTTATCCACATATGAATATCCTCCTTAG	Mutagenesis
treC KO_R	ACTTCTGTAACCACCAGACAGCCTCAAAAGGCCGTAAATTCATGGCACAGAGTGTAGGCTGGAGCTGCTTC	Mutagenesis
cscA KO_F	CAATTCACCAAATTTGCTTAACCAGGATGATTAAAATGACGCAATCTCGATTGCATGGTGTAGGCTGGAGCTGCT	Mutagenesis
KOcscA_R2	TATGTTAACCCAGTAGCCAGAGTGCTCCATGTTGCAGCACAGCCACTCCGTGGGACATATGAATATCCTCCTTAG	Mutagenesis
KOcscB_F	GAATTTTTTAACGACAGGCAGGTAATTATGGCACTGAATATTCCATTCAGAAATGGTGTAGGCTGGAGCTGCTTC	Mutagenesis
KOcscB_R	CCGGTTGAGGGATATAGAGCTATCGACAACAACCGGAAAAAGTTTACGTCTATATCATATGAATATCCTCCTTAG	Mutagenesis
dld_F1	AGCACCCTGCGTCTCGACAAGC	qRT-PCR
dld_R1	CACGACGATCCAATCACCGAGTGC	qRT-PCR
cscA QRT	GTCCGGACATTCCCACATATAG	qRT-PCR
cscA QRT	AGGCAACACGGGGCAGATCCTG	qRT-PCR

* Used for the direct sequencing of Tn*5* insertion sites from purified genomic DNA.

### Generation and screening of Tn5 mutants with altered sucrose utilization phenotypes

A library of ∼10 000 random *E. coli* W mutants was generated using the EZ-Tn5™ <KAN-2> Tnp Transposome™ kit (Epicentre Biotechnologies, WI) as per the manufacturer's instructions to generate transformants containing only one Tn5 insertion. Following transformation, cells were immediately plated onto LB agar containing kanamycin. The library was screened by patching onto M9S2, M9S20 and MacConkey (1% sucrose) plates to identify sucrose utilization mutants. Tn5 insertion sites were identified by direct Sanger sequencing of purified genomic DNA as described previously [33] using primer JSP07 (Table 2) and analyzed by Micromon Sequencing Facility (Clayton, VIC. Australia). To identify nucleotide changes within the *csc* regulon, overlapping PCR products were generated, purified using MinElute PCR purification kit (QIAgen, Doncaster, VIC, Australia) and subsequently sequenced by the Australian Genome Research Facility (Brisbane, QLD Australia) using the Sanger method.

### Site directed chromosomal gene knock-out

Chromosomal gene knock-out was performed using one-step homologous recombination [34], with minor modifications as described previously [18], [35], [36]. Primers used to amplify gene knock-out constructs using pKD3 [34] as template are shown in Table 2. The *cscB* gene is in the same operon as, and immediately downstream from, *cscK*; for this reason, particular care was taken in the primer design to ensure that *cscK* would not be disrupted. Transformants were selected on chloramphenicol and deletion of the target gene(s) was confirmed by PCR amplification across the knock-out locus and subsequent Sanger sequencing of the PCR product (see Table 2 for details of primers used). Where appropriate, the chloramphenicol resistance marker was removed by Flp recombinase as described previously [34], [37]. Precise excision of the chloramphenicol resistance gene was also confirmed by PCR and sequencing. The resulting knock-out strains are listed in Table 1.

### Growth rate analysis

Growth rates were analyzed in a 96-well microtitre plate format as described previously [28] except that cultures were plated onto M9S20 agar and then M9S2 agar prior to pre-culture in M9S2. Growth rates of transposon and knock-out mutants were compared with the wild type *E. coli* W strain and the *cscR* knock-out (WΔ*cscR*) as controls

### qRT-PCR

Mutant and wild-type strains were cultured in M9 minimal media containing 1% glycerol with either 2% or 0.2% sucrose in shake flasks. At mid-log (OD600∼0.4) 10 ml of culture was harvested and immediately resuspended in 1 ml of TRI regent (Invitrogen). Each sample was heated at 70°C for 15 min before storage at −20°C. TRI extraction, DNaseI treatment (Promega) and RNeasy mini column (QIAgen) clean up were performed as per the manufacturer's instructions. PCR was used to confirm complete removal of contaminating genomic DNA from the RNA samples. cDNA was amplified from 600 ng total RNA using Superscript III (Invitrogen) as per the manufacturer's instructions with the following modifications: 50 ng of random hexamer (Invitrogen) was used and the reaction incubated at 50°C for 2.5 hr. An RT− control was performed alongside. RT+ and RT− template was diluted 1/200 with water before use. qRT-PCR was performed in a Corbett Rotor-Gene 3000 using Sybr green UDG mastermix (Invitrogen). Reactions were performed in 15 ul, containing 160 nM each oligo (Table 2) and 2 ul of template. Each assay included (in duplicate): a standard curve generated with genomic DNA (gDNA) for each gene, and triplicate RT+ and duplicate RT− technical replicates for each of three biological replicates. Standard cycling conditions were used and melt curve analysis was performed, confirming only a single product was produced. A standard curve for each gene was determined and the efficiency of the reaction noted. C_t_ values were determined using the default values on the Rotor Gene- 6 software. A correction for gDNA contamination was performed however the levels of gDNA detected were insignificant. The log of the relative abundance value (log R) for each sample was calculated using the equation log*R* = −log*A · C_t_*+log*A*_ref_ *· C_t,_*_ref_ where *A* is the amplification efficiency and *C_t_* is the cycle threshold. The reference gene was *dld* (D-lactate dehydrogenase). To determine statistical significance between log*R* values for each gene and each mutant, a one-way ANOVA was performed, followed by a Tukey's Honest Significant Difference post hoc test.

## Results and Discussion

Sucrose is an important industrial feedstock for bioprocesses [1], [2] and sucrose utilisation is widespread in pathogenic *E. coli* strains [5]. However, significant variation in growth rates on sucrose have been observed between wild-type strains [5] and laboratory strains modified to express the *csc* regulon from EC3132 typically grow slowly when compared to the WT EC3132 strain [11], [27], [28]. These observations suggest that non-*csc* genes might contribute to the sucrose utilisation and/or induction phenotype in *E. coli*. To investigate this, we used *E. coli* W, a non-pathogenic wilt type (WT) strain which uses a *csc* regulon to confer sucrose utilisation [1]. Although *E. coli* W grows as quickly on sucrose, induction of the *csc* regulon requires concentrations ≥1% of sucrose [2], [16].

### Generation and screening of the mutant library

A Tn*5* transposon mutagenesis library was constructed and screened for growth on minimal medium supplemented with either 2% or 0.2% sucrose. From the library of 10,000 kanamycin resistant mutants, 32 mutants with altered sucrose fermentation patterns (improved utilization at low sucrose, or lack of sucrose utilization) were identified. All except one of the strains that could not utilize sucrose had insertions in the *csc* operon (four in *cscB* and three in *cscA*). This supports our earlier studies showing that these two genes are essential for sucrose utilization in wild type *E. coli* W [16]. Previous research has demonstrated that truncation or deletion of the *cscR* gene can confer de-repression at low sucrose [2], [11], [16]; consistent with this, four mutants that could utilize sucrose at 0.2% had insertions in *cscR*. These *csc* mutants were excluded from further studies.

Improved sucrose fermentation can also be attributed to spontaneous mutation events within the *csc* regulon [10], [11]. Frequently, these mutations occur within the repressor or the repressor binding site [10], and lead to de-repression of the regulon, allowing growth on low concentrations of sucrose. To exclude this as a possible reason for improved sucrose utilization, the *csc* regulons of the remaining strains were sequenced. A further ten Tn*5* strains were found to contain spontaneous point mutations in the *csc* regulon. Eight of these were in *cscB*, one in a putative operator binding site within the *csc* promoter region (*cscKB*OP), and one in *cscR* (Q132*) that resulted in truncation of the repressor protein. The mutation in the *cscKB*OP has been observed previously and was shown to confer growth on low sucrose [10], [11]. Interestingly, nearly all of the mutations identified in this study were located within *cscB* despite the fact that only one CscB mutation (Q353H) that allows for growth on low sucrose has been characterized previously [10]. Mutations in *cscB* which improve sucrose transport [10], [11], [38] can also relieve repression, presumably as a consequence of increased concentrations of intracellular sucrose (or fructose and/or glucose) which can then facilitate de-repression. All of these *csc* gene mutants were excluded from further study.

### Deletion of the trehalose repressor gene treR permits growth on low sucrose

Once the *csc* mutants were removed from the study, the Tn*5* insertion sites within the 11 remaining Tn*5* mutants were identified using direct Sanger sequencing of purified genomic DNA (Table 3). The single mutant that was unable to utilize sucrose contained a Tn*5* insertion within *cyaA* (adenylate cyclase), a gene which is essential for cyclic AMP (cAMP) production. Two cAMP-CrpA binding sites are found in the bi-directional *csc* promoter [18], and regulation of both the *cscA* and *cscKB* transcription units by cAMP has been demonstrated previously [10]. In the absence of cAMP, activation of the *csc* regulon by cAMP-CrpA cannot occur; this most likely provides an explanation for why this strain cannot grow on sucrose. Of the remaining mutants, Tn*5* insertions in four mutants were found to disrupt genes associated with DNA replication, recombination and repair. Disruption of these genes may lead to an increased potential for accumulation of mutations elsewhere in the chromosome that we could not identify; for this reason, these strains were not examined further.

**Table 3:** Tn*5* insertion sites in selected non-*csc* insertion/mutation strains.

Strain	Genotype	Suc	Comment	COG group description	COG
Tn2	W *appC::*Tn*5*	+	Cytochrome *bd*-II terminal oxidase subunit	Energy production and conversion	C
Tn9	W *cyaA::*Tn*5*	−	Adenylate cyclase, family 3	Signal transduction	T
Tn32	W *mhpR::*Tn*5 cscB* L363F	+	Mhp operon transcriptional activator	Transcription	K
Tn33	W *mutS::*Tn*5*	+	Methyl-directed DNA mismatch repair protein	DNA replication, recombination and repair	L
Tn54	W *pi349::*Tn*5*	+	phage recombinase	DNA replication, recombination and repair	L
Tn61	W *treR::*Tn*5*	+	HTH-type transcriptional repressor, trehalose PTS uptake operon	Transcription	K
Tn63	W *gspK::*Tn*5*	+	General secretion pathway protein K	Cell motility and secretion	N
Tn65	W *gltS::*Tn*5*	+	Sodium/glutamate symport carrier protein	Amino acid transport and metabolism	E
Tn68	W *hyp::*Tn*5*	+	conserved hypothetical protein	Unknown	S
Tn84	W *truA::*Tn*5*	+	tRNA pseudouridine synthase	Translation, ribosomal structure and biogenesis	J
Tn87	W *treR::*Tn*5*	+	HTH-type transcriptional regulator, trehalose PTS uptake operon	Transcription	K

Strains were grown on M9S2 and M9S20. Sucrose phenotype (Suc) is listed as positive if the strain can grow on low sucrose (0.2%) and negative if it cannot grow on sucrose at all. COG (Clusters of Orthologous Genes) groupings are listed.

Transposon insertions were also found within two transcriptional regulators (two isolates with insertions in *treR* and one with an insertion in *mhpR*) as well as the membrane bound transporters *gltS* and *gspK*. To investigate the role of these genes, three independently isolated site-directed deletion mutants of each of the four targets were constructed in the wild type W strain using homologous recombination. Of the genes targeted, only the *treR* mutants were able to grow on 0.2% sucrose. The growth rate of the WΔ*treR* mutant on 0.2% sucrose was not statistically different from the two original mutant strains (Tn61 and Tn87; ρ≥0.05; Table 4), suggesting that deletion of *treR* is responsible for the phenotype. The lack of growth in the other deletion mutants suggests that the original phenotype observed was not due to the transposon insertion. It is possible that those mutants carry additional mutations which confer the ability to grow on 0. 2% sucrose; the frequency of spontaneous mutations observed in the *csc* regulon even in the absence of selection pressure supports this hypothesis.

**Table 4:** Summary of the growth characteristics of the WΔ*treR* double mutants generated in this study.

	glucose 1%	sucrose 2%			sucrose 0.2%		
	phenotype	phenotype	μ (h^−1^) ± SD	ρ value^1^	phenotype	μ (h^−1^) ± SD	ρ value^2^
W	+++	+++	1.36±0.12	-	−	-	n/a
WΔcsc*R*	+++	+++	1.42±0.06	Ns	++	0.65±0.01	**
Tn61	+++	+++	1.01±0.04	Ns	++	0.19±0.03	ns
Tn87	+++	+++	1.05±0.13	Ns	++	0.28±0.03	ns
WΔtreR	+++	+++	0.99±0.16	ns	++	0.35±0.02	-
WΔtreRΔtreB	+++	+++	1.18±0.33	ns	−	-	n/a
WΔtreRΔtreC	+++	+++	1.29±0.21	ns	++	0.42±0.04	ns
WΔtreRΔcscB	+++	−	-	n/a	ND	-	n/a
WΔtreRΔcscA	+++	−	-	n/a	ND	-	n/a

Strains plated on M9 minimal media supplemented with various carbon sources as indicated in the table. Phenotype, +++ fast growth (sizable colonies in 15 h); ++, growth (small colonies after 15 h); −, no growth after 24 h; ND, not determined. Statistical significance was determined using the Kruskal-Wallis test combined with Dunn's Multiple Comparison test. ρ value^1^, comparison between each sample and W; ρ value^2^, comparison between each sample and WΔ*treR*.

**, ρ value≤0.01;

ns, not significant; n/a, not applicable.

### The trehalose-specific enzyme II (TreB) is required for growth of WΔtreR on low sucrose

**Figure 1:**
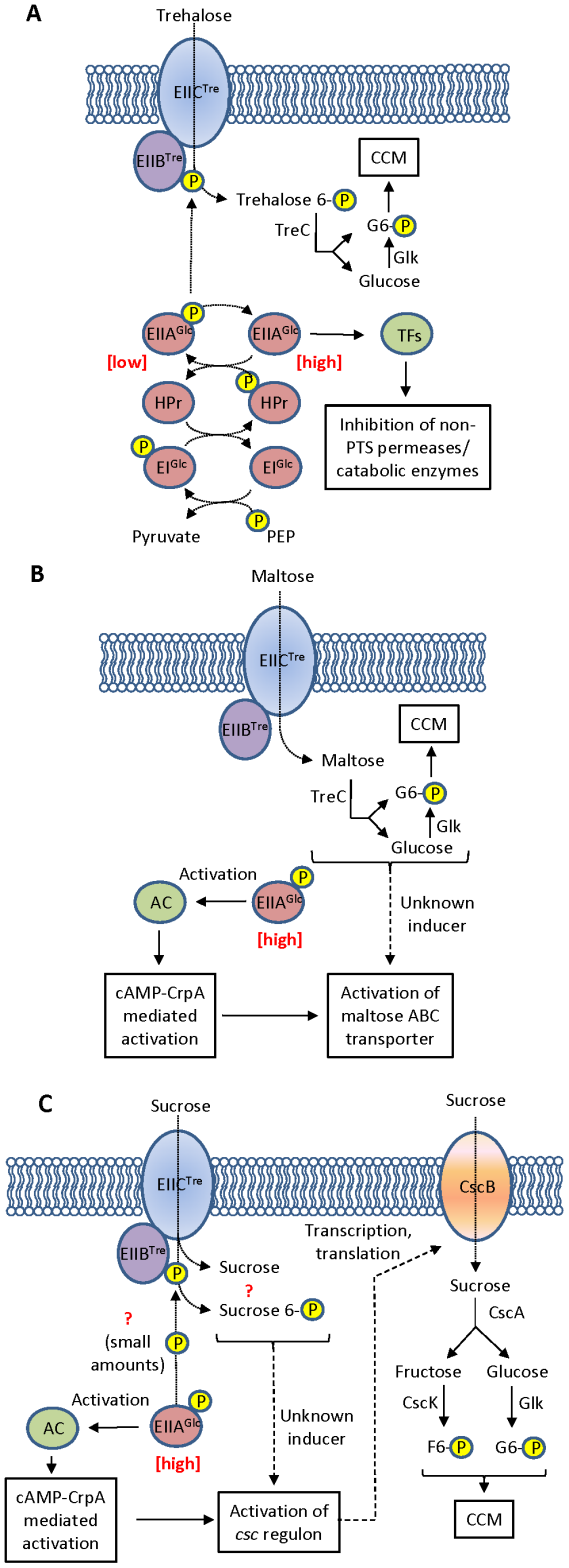
Models for disaccharide transport and utilisation by EIIC^Tre^. In phospho*enol*pyruvate∶carbohydrate transport systems, sugars (in this case, trehalose) are transported with concomitant phosphorylation *via* a PTS-associated phosphorylation cascade (A). For the trehalose PTS, the transporter protein EIIBC^Tre^ consists of a permease (EIIC^Tre^) and a kinase (EIIB^Tre^) domain. EIIB^Tre^ accepts a phosphate group from EIIA^Glc^ in the presence of trehalose transport. EIIC^Glc^ accepts a phosphate from HPr, which in turn accepts a phosphate from the PEP-dependent histidine-protein kinase EI^Glc^, which accepts its phosphate group from phospho*enol*pyruvate. Phosphorylated trehalose is cleaved by TreC to yield 1× phosphorylated (G6P) and 1× unphosphorylated glucose; both of these feed into central carbon metabolism (CCM) (the unphosphorylated glucose is phosphorylated by glucokinase, Glk). In the presence of PTS-mediated sugar transport, the concentration of unphosphorylated EIIA^Glc^ increases; unphosphorylated EIIA^Glc^ transcriptionally inhibits non-PTS permeases and catabolic enzymes that generate internal inducers of the various catabolic regulons (a mechanism known as inducer exclusion) *via* a variety of transcription factors [51]. The trehalose PTS has also been shown to transport maltose [50] (B). In this case, transport is thought to be achieved through facilitated diffusion by EIIC^Tre^ and the maltose not phosphorylated by EIIB^Tre^. In the absence of PTS-mediated phosphorylation, carbon catabolite repression is released: the concentration of phosphorylated EIIA^Glc^ remains high; this activates adenylate cyclase (AC), resulting in an increase in intracellular cAMP concentration; cAMP complexes with the transcription factor CrpA; and cAMP-CrpA transcriptionally activates a wide variety of non-PTS transport systems, including the ABC transporter for maltose. Sucrose transport by TreB (C) may occur with or without concomitant phosphorylation, but most likely occurs without phosphorylation (see text). Transport results in sufficient intracellular sucrose to facilitate induction of the *csc* regulon; phosphorylated EIIA^Glc^ remains high, and the *csc* regulon is activated through cAMP-CrpA. Transported unphosphorylated sucrose may be metabolised *via csc* gene products; phosphorylated sucrose is most likely not metabolised. Once the *csc* genes are induced, sucrose can be imported through the CscB permease and cleaved by the CscA invertase into fructose and sucrose. Fructose is phosphorylated by the CscK fructokinase, and glucose is phosphorylated by Glk; both phosphorylated sugars feed into CCM.

In *E. coli*, uptake of trehalose occurs *via* the phosphoenolpyruvate (PEP)∶carbohydrate phosphotransferase system (PTS) [39], [40]. In this system the trehalose-specific enzyme EIICB^Tre^ (TreB) transports trehalose into the cell with concomitant phosphorylation to deliver trehalose-6-phosphate [41] (see Figure 1A). EIICB^Tre^ does not have a covalently-bound EIIA domain for phosphorylation of the transported sugar; like other non-glucose PTS transporters in the EIIBC domain type, it relies on kinase activity provided by EIIA^Glc^, a component of the primary glucose transport system [41]–[43]. The transported trehalose 6-phosphate is hydrolyzed by TreC to produce glucose and glucose-6-phosphate [39], [44] which are directed into central carbon metabolism. Expression of *treB* and *treC* is activated in the absence of glucose and repressed by TreR in the absence of trehalose-6-phosphate [40].

Substrate plasticity is common in disaccharide transporters [45]–[50]. Functional analysis of the trehalose PTS has shown that maltose can enter the cell *via* the TreB transporter [50] (see Figure 1B). Trehalose is a glucose α(1→1) disaccharide, and maltose is a glucose α(1→4) disaccharide. Interestingly, maltose is transported by facilitated diffusion, and enters the cell in an unphosphorylated state [50]. The substrate flexibility of TreB led us to hypothesize that it might also be able to transport sucrose, a glucose-fructose disaccharide, and that deletion of the *treR* repressor might allow production of TreB at sufficient levels to transport sucrose into the cell to allow for cell growth.

To determine if deletion of the *treR* gene allows for the uptake of sucrose *via* TreB, a double WΔ*treR*Δ*treB* deletion mutant was constructed and tested for growth on 0.2% sucrose. In support of our hypothesis, this mutant was unable to grow on sucrose (Table 4), suggesting that sucrose uptake *via* TreB is essential for the growth of WΔ*treR* on 0.2% sucrose.

### The trehalose hydrolase TreC is not required for growth of WΔtreR on low sucrose

Maltose is thought to be transported by TreB in an unphosphorylated form *via* facilitated diffusion [50]. Maltose is not recognised by the TreC trehalose hydrolase [44], however, transport *via* TreB allows induction of the maltose ABC transporter [50] (see Figure 1B). To determine if hydrolysis by TreC is required for low sucrose utilisation in WΔ*treR*, a double WΔ*treR*Δ*treC* deletion mutant was also constructed. Growth of WΔ*treR*Δ*treC* on 0.2% sucrose (Table 4) was indistinguishable from the WΔ*treR* strain (ρ≥0.05), indicating that, like for maltose, processing of the incoming sucrose by TreC is not required for growth on low concentrations of sucrose. This is consistent with transport studies showing that TreC does not recognise unphosphorylated sucrose [44]; however, there is no information available in the literature for transport or otherwise of phosphorylated sucrose (see comments below).

### cscB is required for the growth of WΔtreR on low sucrose

Based on the results from the WΔ*treR*Δ*treB* mutant, growth on low concentrations of sucrose in the presence of a *treR* mutation relies on uptake of sucrose *via* TreB. To determine if sucrose uptake *via* TreB is sufficient to support growth in a WΔ*treR* background, a double WΔ*treR*Δ*cscB* deletion mutant was constructed and tested for growth on 0.2% and 2% sucrose. WΔ*treR*Δ*cscB* was unable to grow on either concentration (Table 4), demonstrating that *cscB* is also required for growth in WΔ*treR* mutants, and that the uptake of sucrose *via* TreB alone is insufficient for growth.

### Sucrose uptake via TreB de-represses the csc operon allowing for growth on low concentrations of sucrose

**Figure 2:**
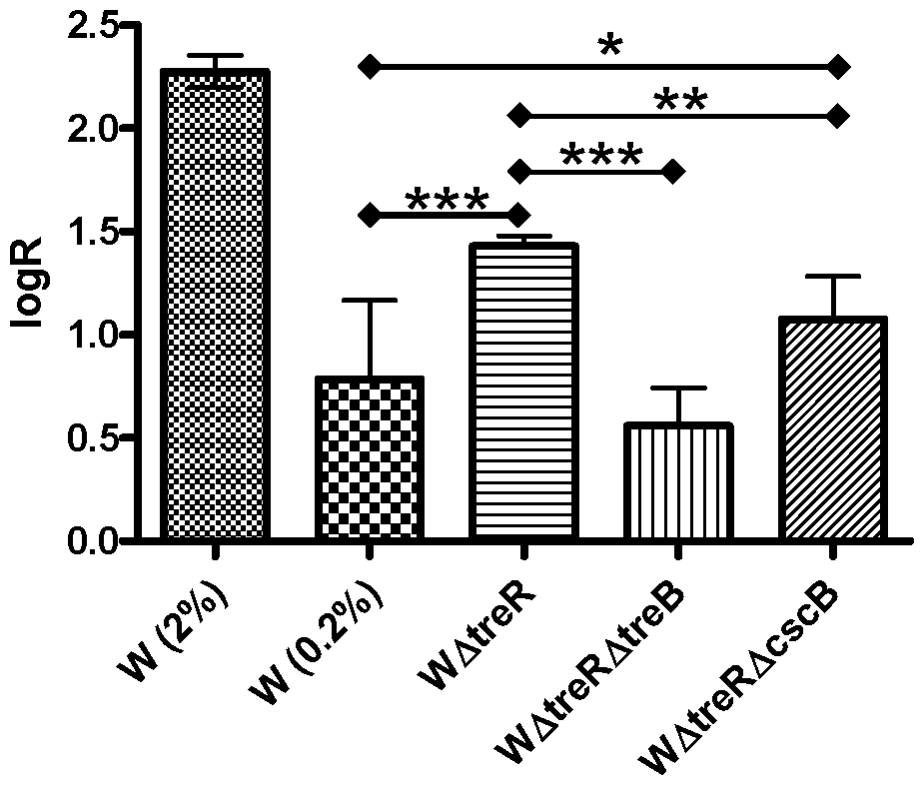
Relative expression of *cscA* in the WΔ*treR* mutants. The expression level of *cscA* was determined by qRT-PCR and log normalized to the level of *dld* (D-lactate dehydrogenase). As a control, W was cultured in minimal media containing 1% glycerol and 2% sucrose. The test strains W, WΔ*treR*, WΔ*treR*Δ*treB* and WΔ*treR*Δ*cscB* were cultured in minimal media containing 1% glycerol and 0.2% sucrose. Statistical significance was determined using one way ANOVA followed by Tukey's HSD test. The average relative expression was determined from three independent mutants (or three biological replicates of W). Error bars are SEM; * p≤0.05, ** p≤0.01, *** p≤0.001.

Based on the results from the mutagenesis studies, growth of the WΔ*treR* mutant in 0.2% sucrose relies on the uptake of sucrose *via* TreB to induce the *csc* regulon. We hypothesised that sucrose transported by TreB, while insufficient to support growth, can trigger de-repression of the *csc* operon, allowing for uptake of sucrose *via* CscB and subsequent processing of the sucrose into glucose and fructose by CscA. To confirm this hypothesis, QRT-PCR was performed on W, WΔ*treR*, WΔ*treR*Δ*treB* and WΔ*treR*Δ*cscB* cultured on minimal media containing 1% glycerol supplemented with 0.2% sucrose (Figure 2). Glycerol was included to support the growth of strains that would otherwise not grow on sucrose alone. The *cscA* gene was used as a reporter of transcription. The wild-type strain with 2% sucrose was included as a positive control for *cscA* expression under induced conditions.

As expected, expression of *cscA* was strongest in the WT strain under strongly inducing conditions (2% sucrose). Expression was also observed at a lower level on 0.2% sucrose, even though 0.2% sucrose as a sole carbon source is insufficient to support growth of this strain. This is not surprising: in the absence of sucrose, a basal level of expression (leaky expression) from the *csc* regulon is expected as this allows for the cell to detect sucrose should it become available. In the WΔ*treR* mutant, the expression of *cscA* was significantly higher than that of the WT control cultured under the same conditions (ρ≤0.001), indicating that induction the *csc* regulon occurs when this mutant is cultured on 0.2% sucrose. The degree of induction in the W*ΔtreR* mutant was less than that of W cultured in 2% sucrose; this may be due to the low sucrose transport rates of TreR resulting in inducer concentrations that are insufficient for full deactivation of CscR.

In the double deletion strain WΔ*treR*Δ*treB* in the presence of 0.2% sucrose, expression of *cscA* returns to levels that are statistically indistinguishable (ρ≥0.05) from the WT on 0.2% sucrose, providing additional evidence that sucrose uptake *via* TreB is essential for growth of the WΔ*treR* mutant on 0.2% sucrose. In the final double deletion strain WΔ*treR*Δ*cscB*, expression of *cscA* was increased compared to W when grown in the presence of 0.2% sucrose (ρ≤0.05), even though the ability to grow on sucrose as a sole carbon source is lost. This result demonstrates that induction of the *csc* regulon in WΔ*treR*Δ*cscB* occurs in the absence of sucrose uptake *via* CscB. Importantly, expression of *cscA* in the WΔ*treR*Δ*cscB* mutant was lower than that observed in the WΔ*treR* mutant (ρ≤0.01). This finding indicates that sucrose uptake *via* CscB does contribute to the level of induction observed in the original strain. We hypothesize that the increased induction observed in WΔ*treR* is the result of feedback in which the regulon is initially induced by sucrose transported by TreB, and subsequent expression of the *csc* regulon and uptake of sucrose *via* CscB results in further induction.

### Sucrose transport by TreB: phosphorylated or unphosphorylated?

A question that remains is whether sucrose is transported in a phosphorylated form, like trehalose [41], [42] (Figure 1A), or unphosphoryated, as maltose is thought to be [50] (Figure 1B). One possible explanation for the lack of growth in the WΔ*treR*Δ*cscB* mutant is that the sucrose is phosphorylated and cannot be metabolized (Figure 1C); this is plausible given that the W genome does not encode a sucrose-6-phosphate hydrolase [1]. The phosphorylated sucrose might still act as an inducer of the *csc* regulon, allowing transport of sucrose *via* CscB. Under this scenario, phosphorylated EIIA^Glc^ remains at relatively high concentrations, since transport levels are low. Phosphorylated EIIA^Glc^ activates adenylate cyclase, increasing cellular cAMP concentrations. An increase in cellular cAMP would allow release of catabolite repression *via* the cAMP-CrpA transcriptional activator, activating the *csc* regulon (which responds to cAMP [10]; see Figure 1C). Thus, cellular conditions would be permissive for transcription of the *csc* regulon. However, it is known that transport rates of EII enzymes that can carry out facilitated diffusion increase significantly when they are phosphorylated [51]. EII enzyme phosphorylation is linked to carbohydrate phosphorylation; consequently, one would expect transport rates to increase, and potentially be sufficient to support growth. This situation occurs in PTS-mediated sensing of carbon sources for PTSs that can transport unphosphorylated carbohydrates, and provides a mechanism for rapid upregulation of transport in the presence of preferred substrates. Feedback of this cycle results in increased transport rates, more phosphorylation of EIIB^Tre^, and decreased concentrations phosphorylated EIIA^Glc^. In this case, that would ultimately result in repression of the *csc* regulon (see Figure 1A and 1C). Furthermore, the increase in unphosphorylated EIIA^Glc^ is likely to result in transcriptional inhibition of the *csc* regulation (‘inducer exclusion’; [51]) (see Figure 1A). Clearly, however, this does not happen: the *csc* regulon is instead induced, and the requirement of CscB demonstrates that transport of sucrose by EIIB^Tre^ remains at very low levels. Transport of phosphorylated sucrose is therefore only a likely mechanism if phosphorylation of EIIB^Tre^ does not significantly enhance transport of sucrose.

Alternatively, sucrose might be transported in an unphosphorylated state (Figure 1C). Under this scenario, the unphosphorylated sucrose facilitates induction of the *csc* regulon and EIIA^Glc^ remains high, allowing cAMP-CrpA mediated de-repression in concert with induction (Figure 1C). The sucrose is cleaved by CscA, and the resulting hexoses are phosphorylated by CscK/Glk before feeding into central carbon metabolism. Transport of sucrose by EIIB^Tre^ remains at very low rates and, in the absence of CscB, is insufficient to support growth. This scenario most easily explains the observed experimental data and is consistent with currently-understood models of PTS-mediated transport.

### Conclusion

Direct demonstration of sucrose transport by TreB using available methods is technically challenging due to the very low levels of sucrose transported. However, the mutant analysis presented here provides compelling evidence that TreB can transport sucrose, albeit at very low concentrations. We also showed that the transported sucrose is sufficient to facilitate induction of the *csc* operon. Interestingly, a very similar association is shown between the trehalose transporter and maltose catabolism, controlled in this case by an ABC transporter [50]. TreB is also highly homologous to the EII^Scr^ of the sucrose-specific PTS encoded on the pUR400 plasmid of enteric bacteria [42], which can also transport maltose and lactose [52]. Maltose porins can also transport sucrose and trehalose efficiently [53]. Furthermore, CscB in fact transports maltose at a higher affinity than sucrose [45]. These observations suggest that the substrate plasticity shared by the enzymes might be related to their structural similarity, and that trehalose/sucrose/maltose might share common biochemical properties from a transport point of view. As is the case with direct demonstration of transport in the first instance, direct demonstration of what form the sucrose is transported in is technically challenging due to the low levels transported, and this question remains unanswered. Regardless, to our knowledge, this is the first time that TreB has been shown to transport sucrose.
